# Impact of Bacterial and Human Genetic Variation on *Staphylococcus aureus* Infections

**DOI:** 10.1371/journal.ppat.1005330

**Published:** 2016-01-14

**Authors:** Julia A. Messina, Joshua T. Thaden, Batu K. Sharma-Kuinkel, Vance G. Fowler

**Affiliations:** 1 Division of Infectious Diseases, Department of Medicine, Duke University, Durham, North Carolina, United States of America; 2 Duke Clinical Research Institute, Duke University, Durham, North Carolina, United States of America; University of North Carolina at Chapel Hill School of Medicine, UNITED STATES

The clinical diversity of syndromes caused by *Staphylococcus aureus* arises from a complex interplay between host and pathogen. Genetic variation can result in increased susceptibility to infection within the host and an increased capacity for virulence within the pathogen, resulting in a wide array of clinical syndromes. This review presents evidence for the role of bacterial and human genetic variation in influencing the clinical outcome of *S*. *aureus* infections.

## What Role Does Bacterial Genetic Variation Play in *S*. *aureus* Infections?

Genetic variation that encodes for virulence, antibiotic resistance, and host adaptation can be introduced through horizontal transfer of mobile genetic elements (MGE)—including bacteriophages, pathogenicity islands (SaPI), plasmids, transposons, and cassette chromosomes—between *S*. *aureus* isolates [1]. MGE tend to distribute asymmetrically within *S*. *aureus* isolates of the same genetic background, or clonal complex (CC). Staphylococcal toxic shock syndrome, a disease of unchecked inflammatory cascade induced by superantigen toxic shock syndrome toxin-1 (TSST-1), provides a classic example of the potential impact of MGE on clinical virulence of *S*. *aureus*. The gene that encodes for TSST-1, *tst*, is located on the MGE SaPI1 and is spread horizontally in distinct CCs of clinical *S*. *aureus* isolates [2,3].

Asymmetric clustering of adhesins and toxins within specific *S*. *aureus* CCs may be associated with an increased risk for other types of *S*. *aureus* infection. *S*. *aureus* from CC5 and CC30 genotypes were significantly associated with hematogenous complications, including left-sided native valve infective endocarditis (IE), when compared with clinical *S*. *aureus* isolates of other genotypes from the same referral area [4]. The association between CC30 and IE was also found in an international collection of geographically matched *S*. *aureus* isolates from patients with either left-sided IE or soft tissue infection [5]. In rabbit models, *S*. *aureus* isolates belonging to the USA200, a genotype defined by pulsed-field gel electrophoresis that approximately corresponds to CC30 in the multi-locus sequence typing strategy, were significantly more likely to cause IE but less likely to cause lethal sepsis than isolates from either USA300 (CC8) or USA400 (CC1) genetic backgrounds [6]. The attenuated sepsis virulence of CC30 has also been documented in murine models [7–9].

What makes the CC30 clonotype distinct? The reduced sepsis virulence of CC30 is at least partially explained by a stop codon mutation in *hla*, the gene in *S*. *aureus* that encodes for the potent virulence factor alpha toxin [7]. A number of other genes also appear to be expressed differently in CC30 isolates. Using in vitro RNA sequencing, clinically derived CC30 *S*. *aureus* differed from isolates of other lineages by significantly higher expression of protein A (*spa*), putative membrane proteins (SAR2274, SAR2275), and exported proteins (SAR2016, SAR0437, and SAR0694), as well as significantly lower expression levels of genes within the pyrimidine biosynthesis pathway (*carB*, *pyrC*, *pyrR*, *pyrE*, and *pyrF*), iron repressible ABC transport (SAR0641, SAR0642, and SAR0643), and an azoreductase (*acpD*) [9]. Recently, CC30 was also shown to express an allelic variant of the key toxin Phenol-Soluble Modulin α3 that conferred reduced chemotactic potential and increased hematogenous seeding [8]. Which of these differences, if any, contribute to CC30’s association with specific clinical syndromes is an area of ongoing investigation.

Bacterial genetic variation can also occur on the level of polymorphisms within specific genes that contribute to the virulence of *S*. *aureus*. For example, fibronectin-binding protein A (FNBPA), encoded by *fnbA*, is thought to play a critical role in the initiation of IE [10]. FNBPA binds to human fibronectin, a protein that deposits on sites of endothelial disruption as well as the endovascular leads of permanent pacemakers and defibrillators. We evaluated the possibility that genetic variation within the binding regions of *fnbA* of *S*. *aureus* bloodstream isolates would be associated with an increased risk of cardiac device infection (CDI) in patients who developed *S*. *aureus* bacteremia [11]. Three nonsynonymous single nucleotide polymorphisms (SNPs), E652D, H782Q, and K786N, in the binding region of *fnbA* of bloodstream *S*. *aureus* isolates, were significantly associated with an increased risk of CDI in the source patient. Using atomic force microscopy (AFM), isolates containing these SNPs exhibited significantly higher frequency and strength of binding to fibronectin. Synthesized peptides containing two of the three polymorphisms (H782Q and K786N double mutant) exhibited 34% higher binding activity than the wild type by AFM. In silico molecular dynamics simulations demonstrated that residues of each of the three polymorphisms in FNBPA formed extra hydrogen bonds with fibronectin, providing a potential explanation for this observation of higher binding affinity. The association between specific *fnbA* SNPs and an increased risk of CDI was recently validated in a cohort of German patients with cardiac devices and *S*. *aureus* bacteremia [12]. Interestingly, however, no similar association was seen in patients with prosthetic joints and *S*. *aureus* bacteremia [13]. The apparent specificity of association between *fnbA* SNPs and infection type may be due in part to the fact that arthroplasties lack a fibrin sheath, the fibronectin-rich coating present on endovascular leads of cardiac devices [14].

## What Is the Role of Host Genetic Variation in *S*. *aureus* Infections?

Host genetic characteristics can also influence the host–pathogen interaction (Table 1). Higher rates of *S*. *aureus* infections have been observed in genetically distinct ethnic populations [15–18]. Patients with rare genetic disorders such as Chédiak-Higashi syndrome [19], Hyper-IgE syndrome [20], IRAK-4 deficiency [21], MyD88 deficiency [22], and chronic granulomatous disease [23] also exhibit susceptibility to *S*. *aureus* infection. Finally, different strains of sheep [24], cattle [25], and mice [26] have different susceptibility to *S*. *aureus* infection, sepsis, and death.

**Table 1:** Evidence for genetic variation and *Staphylococcus aureus* infection.

	Evidence for Variation
**Host**	**Higher *S*. *aureus* infection rates in genetically distinct populations of humans:**
	• African Americans [15]
	• New Zealand Maori [17]
	• Australian Aboriginals [16]
	• Canadian Aboriginals [18]
	**Increased susceptibility to *S*. *aureus* infection in rare genetic disorders:**
	• Chédiak-Higashi syndrome [19]
	• Hyper-IgE syndrome [20]
	• IRAK-4 deficiency [21]
	• MyD88 deficiency [22]
	• Chronic granulomatous disease [23]
	**Increased susceptibility to *S*. *aureus* infection in animals:**
	• Cattle [25]
	• Inbred mice [26]
	• Sheep [24]
	**Human genotyping studies:**
	• Nelson et al. (2014): 361 cases of healthcare-associated *S*. *aureus* bacteremia and 699 hospitalized controls [28]
	• Ye et al. (2014): 309 cases of *S*. *aureus* infection and 2,925 controls [39]
	• DeLorenze et al. (2015): 4,701 cases of *S*. *aureus* infection and 45,052 matched controls [29]
**Pathogen**	**Clonal Variation:**
	• CC5 and CC30 associated with hematogenous complications [4]
	• CC30 more likely to cause IE [5]
	• CC22 MRSA with high vancomycin MIC more likely to cause hematogenous complications such as IE from catheter-related bloodstream infections [40]
	• USA200 isolates (CC30) caused more IE but less lethal sepsis than USA300 (CC8) or USA400 (CC1) [6]
	**Gene-Level Variation:**
	• Gene for toxic shock toxin TSST-1 carried by SaPI [2]
	• Staphylococcal scalded skin syndrome is associated with exfoliative toxin gene *etb* whereas *eta* is associated with bullous impetigo [41]
	**Single Nucleotide Variation:**
	• Polymorphisms in *fnbA* SNPs associated with cardiac device infection in patients with *S*. *aureus* bacteremia [11,12]

Clonal complex (CC)

Infective endocarditis (IE)

Methicillin-resistant *Staphylococcus aureus* (MRSA)

Minimal inhibitory concentration (MIC)

*Staphylococcus aureus* pathogenicity islands (SaPI)

Single nucleotide polymorphism (SNP)

Despite this indirect evidence, none of the handful of studies published to date have confirmed the role of human genetic variation in *S*. *aureus* colonization and infection. In a study of adult Danish twins, investigators report *S*. *aureus* nasal carriage in 26.3% of the 617 twin pairs studied, with a concordance rate among monozygotic twins only slightly greater than the overall prevalence [27]. No sign of heritability was observed, and concordance did not vary based upon monozygotic or dizygotic lineage or gender.

Three genome-wide association studies (GWAS) have looked at potential associations between common genetic variants and human susceptibility to *S*. *aureus* infection. Nelson et al. used a GWAS approach to compare 361 Caucasian patients with healthcare-associated *S*. *aureus* bacteremia (SAB) to 699 hospitalized controls without *S*. *aureus* infection [28]. No genome-wide significant common variant was found to be associated with risk of acquiring SAB or severity of SAB (Bonferroni correction, *p* < 9.2x10^-8^). However, upon excluding the interaction between host SNP and bacterial CC, the investigators did note that rs2043436, an SNP located on the candidate gene *CDON*, which encodes a cell surface receptor that is a member of the immunoglobulin family, was associated with severity of infection at the level of *p* = 1.64x10^-6^. Ye et al. (2014) used GWAS to compare 309 cases with *S*. *aureus* infection to 2,925 uninfected adult Northern European control subjects. Again, none of the SNPs identified met genome-wide significance (*p* < 5x10^-8^). Four SNPs approached significance at a level of *p* < 10^−5^. Genes associated with these SNPs were *PDE4B* (rs2455012), involved in bacterial-induced inflammation; *TXNRD2* (rs3804047), involved in the maintenance of thioredoxin in a reduced state; *VRK1*, which encodes a serine and/or threonine kinase; B*CL11B* (rs7152530), which encodes a repressor involved in T cell development; and *PNPLA5* (rs470093), involved in autophagosome function.

Most recently, DeLorenze et al. provide the first GWAS evidence of human genetic susceptibility to *S*. *aureus* infection. The investigators genotyped a Caucasian population of 4,701 cases of *S*. *aureus* infection and 45,344 matched controls [29]. Two imputed SNPs (rs115231074: *p* = 1.3 x 10^−10^ and rs35079132: *p* = 3.8 x 10^−8^) achieved genome-wide significance, and one adjacent genotyped SNP was nearly significant genome-wide (rs4321864: *p* = 8.8 x 10^−8^). These polymorphisms were located near HLA-DRA and HLA-DRB1 genes on chromosome 6 in the HLA class II region. Significant evidence supports the possibility that HLA class II haplotypes may influence human susceptibility to *S*. *aureus* infection. First, specific HLA haplotypes (HLA II DR14 and/or DQ5) are associated with susceptibility to invasive *Streptococcus pyogenes* infection in patients [30] and determine severity of response to bacterial superantigens from both *S*. *pyogenes* [31] and *S*. *aureus* [32]. Second, *S*. *aureus* superantigens, including TSST-1, bind to the HLA II DR1 molecule [33] and are critical in the development of *S*. *aureus* bacteremia and endocarditis [34]. Third, nasal carriage of *S*. *aureus* is associated with the HLA-DR3 and HLA-DR7 class II serotypes [35]. Finally, polymorphisms in HLA-DRB1 are strongly associated with rheumatoid arthritis [36], an inflammatory disease characterized by a high risk of *S*. *aureus* infection.

## What Are the Future Directions in the Study of Genetic Variation and *S*. *aureus* Infection?

Studying the impact of bacterial genetic variation on infection severity in patients will improve our understanding of pathogenesis and will ultimately inform vaccine development and future therapeutic targets. Similarly, insights into the role of human genetic variation on invasive *S*. *aureus* infection will identify high-risk populations in whom expensive and invasive diagnostic and therapeutic strategies can be invested in an increasingly cost-conscious healthcare environment. Achieving these potential advances, however, will require overcoming a number of scientific and practical limitations. First, virulence in *S*. *aureus* is noteworthy for its redundancy, with many proteins exhibiting overlapping function. For example, at least four *S*. *aureus* proteins have the capacity to bind fibrinogen: FNBPA, clumping factor A, clumping factor B, and bone sialoprotein-binding protein [37]. At least two of these proteins, FNBPA and fibronectin-binding protein B, also bind to fibronectin. Next, specific bacterial genes are likely to only be relevant in certain types of infection. For example, genes involved in infections initiated by bacterial binding of host tissues (e.g., IE and osteoarticular infections) are likely to differ from those involved in toxin-mediated syndromes (e.g., toxic shock syndrome, staphylococcal scalded skin syndrome, and necrotizing fasciitis). Finally, genome sequencing of isolates causing invasive disease has shown considerable within-host diversity in *S*. *aureus*, including multiple mutations in the same genes [38]. This within-host diversity may rise and fall over time and be biologically relevant, resulting in inactivation of global virulence regulators and changes in phage copy number.

Given these multiple sources of potential confusion, translational investigations focused on staphylococcal pathogenesis should strictly minimize sources of study variation. Bacterial genetic variation could be reduced by limiting studies to infections caused by a specific genotype of *S*. *aureus*. Variation introduced by the inclusion of multiple infection types (e.g., IE versus pneumonia versus soft tissue infection) can be reduced by focusing on a single, carefully defined clinical syndrome. For example, focusing on complementary bacterial receptors and host ligands, such as *S*. *aureus* FNBPA and human fibronectin in patients with hematogenous cardiac device-associated infections, reduces the possibility of a false negative result by minimizing the number of host–pathogen interactions at play. Finally, human genetic variation may be minimized by limiting study populations to single ethnic backgrounds when conducting human genotyping studies.

In conclusion, substantial evidence for the impact of genetic variation on susceptibility to *S*. *aureus* infection exists. Within the pathogen, evidence is found at the clonal, gene, and SNP levels. Observational studies of genetically distinct ethnic populations and inbred animals also suggest the importance of host genetic variation on the initiation and severity of *S*. *aureus* infection. More translational studies investigating the role of host genetic variability in *S*. *aureus* infection are warranted. The confounding impact of heterogeneity introduced into genetic association studies in *S*. *aureus* can be minimized by limiting study populations by infection type, pathogen genotype, and host ethnicity.
